# Biochemical, sex hormonal, and anthropometric predictors of non-alcoholic fatty liver disease in polycystic ovary syndrome

**DOI:** 10.1186/s12905-025-03648-9

**Published:** 2025-03-14

**Authors:** Xintong Li, Min Min, Fangfang Duan, Xiangyan Ruan, Li Xu

**Affiliations:** 1 Department of Radiology, Beijing Jishuitan Hospital, Capital Medical University, Beijing, 100035 PR China; 2 Department of Gynecological Endocrinology, Beijing Obstetrics and Gynecology Hospital, Capital Medical University, Beijing Maternal and Child Health Care Hospital, Beijing, 100026 PR China; 3 Department of Gynecology, Aviation General Hospital, Beijing, China; 4 Clinical Epidemiology Research Center, Beijing Jishuitan Hospital, Capital Medical University, Beijing, China

**Keywords:** Polycystic ovary syndrome, Non-alcoholic fatty liver disease, Overweight, Obesity, Metabolic syndrome, Sex hormones

## Abstract

**Background:**

Polycystic ovary syndrome (PCOS) is linked to non-alcoholic fatty liver disease (NAFLD). Biochemical, sex hormonal, and anthropometric indicators have been explored for screening NAFLD in PCOS patients. However, the accuracy of NAFLD screening using these indicators in PCOS patients remains uncertain. This study aimed to identify biochemical, sex hormonal, and anthropometric indicators associated with NAFLD in overweight and obese PCOS patients and assess the diagnostic efficacy of combined indicators.

**Methods:**

This cross-sectional study (Clinical trial number ChiCTR1900020986; Registration date January 24th, 2019) involved 87 overweight or obese women with PCOS (mean age 29 ± 4 years). Measurements included anthropometric indices, biochemistry, sex hormone levels, and liver proton density fat fraction (PDFF). Correlation analysis, intergroup comparisons, and logistic regression analysis were used to identify risk factors for NAFLD (PDFF > 5.1%). The receiver operating characteristic curve, area under the curve (AUC), sensitivity, specificity, positive predictive value, and negative predictive value were used to determine cut-off values and evaluate diagnostic accuracy.

**Results:**

Liver PDFF was 7.69% (3.93%, 14.80%) in overweight and obese PCOS patients, with 67.8% diagnosed with NAFLD. NAFLD was associated with increased body mass index (BMI), abdominal circumference (AC), and triglyceride, total cholesterol (TC), low-density lipoprotein-cholesterol (LDL-C), glucose, insulin, and free testosterone (FT) levels, and with decreased high-density lipoprotein-cholesterol (HDL-C) and sex hormone-binding globulin (SHBG) levels (*P* < 0.05). Risk factors for NAFLD in PCOS included BMI > 26.8 kg/m^2^, AC > 88.3 cm, triglyceride > 1.57 mmol/L, TC > 4.67 mmol/L, LDL-C > 3.31 mmol/L, glucose > 4.83 mmol/L, insulin > 111.35 pmol/L, FT > 7.6 pg/mL and SHBG < 25 nmol/L (*β* = 1.411–2.667, *P* < 0.005). A multi-indicator model including triglycerides, LDL-C, glucose, insulin, and SHBG showed higher diagnostic accuracy (AUC = 0.899, *P* < 0.001) for screening NAFLD in PCOS patients than single indicators (AUC = 0.667–0.761, *P* < 0.05).

**Conclusions:**

Overweight and obese PCOS patients have higher incidences of liver PDFF and NAFLD. A multi-indicator model including triglycerides > 1.57 mmol/L, LDL-C > 3.31 mmol/L, glucose > 4.83 mmol/L, insulin > 111.35 pmol/L, and SHBG < 25 nmol/L is highly accurate for screening NAFLD in overweight and obese PCOS patients.

## Introduction

Polycystic ovary syndrome (PCOS) is a highly prevalent endocrine disorder among women of reproductive age. The worldwide prevalence ranges from 4 to 21% depending on the diagnostic criteria applied [1]. PCOS is characterised by irregular menstrual cycles, biochemical and/or clinical hyperandrogenism, ovulatory dysfunction, and polycystic ovarian morphology observed during ultrasonographic examination [1, 2]. Epidemiological data indicate a close relationship between PCOS and obesity, manifested by a high prevalence of overweight or obesity (38–88%) [3]. Conversely, overweight or obese women have a significantly increased risk of developing PCOS compared to their non-obese counterparts, with an odds ratio of 2.77, as previously reported [4]. Obesity exacerbates many adverse outcomes of PCOS, resulting in more severe metabolic and reproductive abnormalities in obese women with PCOS than in those without obesity [5, 6].

PCOS is also highly associated with non-alcoholic fatty liver disease (NAFLD) [7], with a prevalence ranging from 34 to 70% among women with PCOS [8]. NAFLD is characterised by excessive and pathological fat accumulation within hepatocytes (hepatic steatosis) in the absence of significant alcohol consumption [9] and represents a spectrum of liver damage, including non-alcoholic fatty liver (NAFL), non-alcoholic steatohepatitis (NASH), advanced fibrosis, and cirrhosis [10]. The gold standard for diagnosing NAFLD is a liver biopsy [11], with the diagnostic criterion being fat content exceeding 5%. However, a liver biopsy is an invasive procedure that cannot be performed in all patients. Chemical shift-encoded (CSE) magnetic resonance imaging (MRI) proton density fat fraction (PDFF) is regarded as the most accurate non-invasive technique for quantifying liver fat content in vivo [12] and has been shown to be highly consistent with the results derived from histological and chemical methods [13]. NAFLD can be diagnosed when the fat content of the PDFF exceeds 5.1% [14].

The link between PCOS and NAFLD has been established along with other risk factors, such as obesity, insulin resistance (IR), dyslipidaemia, diabetes mellitus, and metabolic syndrome [10, 15, 16]. Both PCOS and NAFLD are related to anthropometric indicators as well as biochemical and hormonal abnormalities [17]. In addition to liver biopsy and MRI, several studies have attempted to screen for NAFLD in patients with PCOS using biochemical and sex hormonal indicators such as the free androgen index (FAI), sex hormone-binding globulin (SHBG), alanine aminotransferase, and lipid accumulation product [18, 19]. However, the accuracy of NAFLD screening using these indicators in PCOS patients remains uncertain. Therefore, this study aimed to explore the indicators associated with NAFLD in overweight and obese young patients with PCOS and investigate the diagnostic efficacy of combined indicators.

## Methods

### Participants

All participants and the program implementation were derived from the Beijing Municipal Administration of Hospitals’ Ascent Plan (No. DFL20181401). This study was approved by the Regional Ethics Committee of Beijing Obstetrics and Gynecology Hospital, Capital Medical University (Protocol number 2018-ky-011-01; Clinical trial number ChiCTR1900020986; Registration date January 24th, 2019) and conducted in accordance with the principles of the Declaration of Helsinki. Written informed consent was obtained from all participants prior to their participation in the study.

Overall, 87 overweight or obese women with PCOS were recruited from the Division of Reproductive Endocrinology and Metabolism between February and December 2019. The enrolment criteria were Chinese women aged 16–45 years with PCOS and a body mass index (BMI) ≥ 24 kg/m^2^. Overweight and obesity were defined as BMI 24.0–27.9 kg/m^2^ and BMI ≥ 28.0 kg/m^2^, respectively [20]. The diagnosis of PCOS was based on the Chinese Diagnostic Criteria and Guidelines of 2018 [21], referring to the Rotterdam Criteria of 2003 [22]. Oligomenorrhea, amenorrhoea, or irregular uterine bleeding were essential for diagnosis and had to meet one of the following two criteria: (1) clinical or biochemical hyperandrogenism and (2) polycystic ovarian morphology on ultrasound examination (12 or more follicles of 2–9 mm in unilateral or bilateral ovaries, and/or an ovarian volume of more than 10 mL). PCOS was diagnosed after excluding other diseases that may cause excess androgen and disordered ovulation, such as Cushing’s syndrome, nonclassical congenital adrenal hyperplasia, hyperprolactinemia, functional hypothalamic amenorrhoea, premature ovarian insufficiency, thyroid disorders, and androgen-secreting tumours. Additionally, patients were excluded if they had a history of viral and autoimmune liver diseases, drug-induced liver disease, hepatic lobectomy, focal changes in the liver, or alcohol consumption exceeding 20 g/day.

### Anthropometric evaluation

Anthropometric evaluations included measurements of weight (kg), height (m), and abdominal circumference (AC, cm). Weight (kg) and height (m) were measured using a standard height-weight scale, and BMI was calculated as weight/height^2^ (kg/m^2^).

### Biochemistry and sex hormonal measurements

Biochemical profiles included triglycerides (mmol/L), total cholesterol (TC, mmol/L), high-density lipoprotein cholesterol (HDL-C, mmol/L), low-density lipoprotein cholesterol (LDL-C, mmol/L), fasting plasma glucose (mmol/L), and fasting insulin (pmol/L) levels. Sex hormone measurements included follicle-stimulating hormone (FSH, IU/L), luteinising hormone (LH, IU/L), oestradiol (pg/mL), progesterone (ng/mL), total testosterone (TT, pg/mL), free testosterone (FT, pg/mL), prolactin (ng/mL), and SHBG (nmol/L). The LH to FSH ratio was calculated as LH (IU/L) / FSH (IU/L).

All blood samples were collected in the early morning after an overnight fast for patients with PCOS and amenorrhoea exceeding 3 months and between days 2 and 4 of the menstrual cycle for patients who ovulated. All laboratory assays were performed in the clinical laboratory of the Beijing Obstetrics and Gynecology Hospital, Capital Medical University. Biochemical indicators were measured using a Synchron LX-20 automated analyser (Beckman Coulter, CA, US). Sex hormone and insulin levels were determined by chemiluminescence immunoassay using an ADVIA Centaur XP automated analyser (Siemens Healthcare Diagnostics, CA, US).

### MRI protocol and measurement

On the day of blood collection, CSE-MRI was performed at the Beijing Jishuitan Hospital, Capital Medical University (Beijing, China). The CSE-MRI protocol [23] and liver fat measurement methods [24] have been described in detail in our previous studies. All patients underwent MRI using a 3.0-T MRI system with a 32-channel torso body coil (Ingenia, Philips Healthcare, Best, the Netherlands). A 3D spoiled gradient-echo sequence was acquired in a single breath-hold using multiple acquired echoes to generate fat, water, T2^*^, and R2^*^ images. In-phase and opposed-phase images were synthesised from the water-fat images. The following scan parameters were used: repetition time (ms)/echo time 1 (ms), 9.1/ 1.33; six echoes with an echo time shift of 1.3 ms; flip angle, 3º; two signal averages; field of view, 360 × 330 × 120 mm^3^; voxel size, 2.5 × 2.5 × 3.0 mm^3^; sensitivity encoding, 2; and imaging time, 12.5 s.

**Fig. 1:**
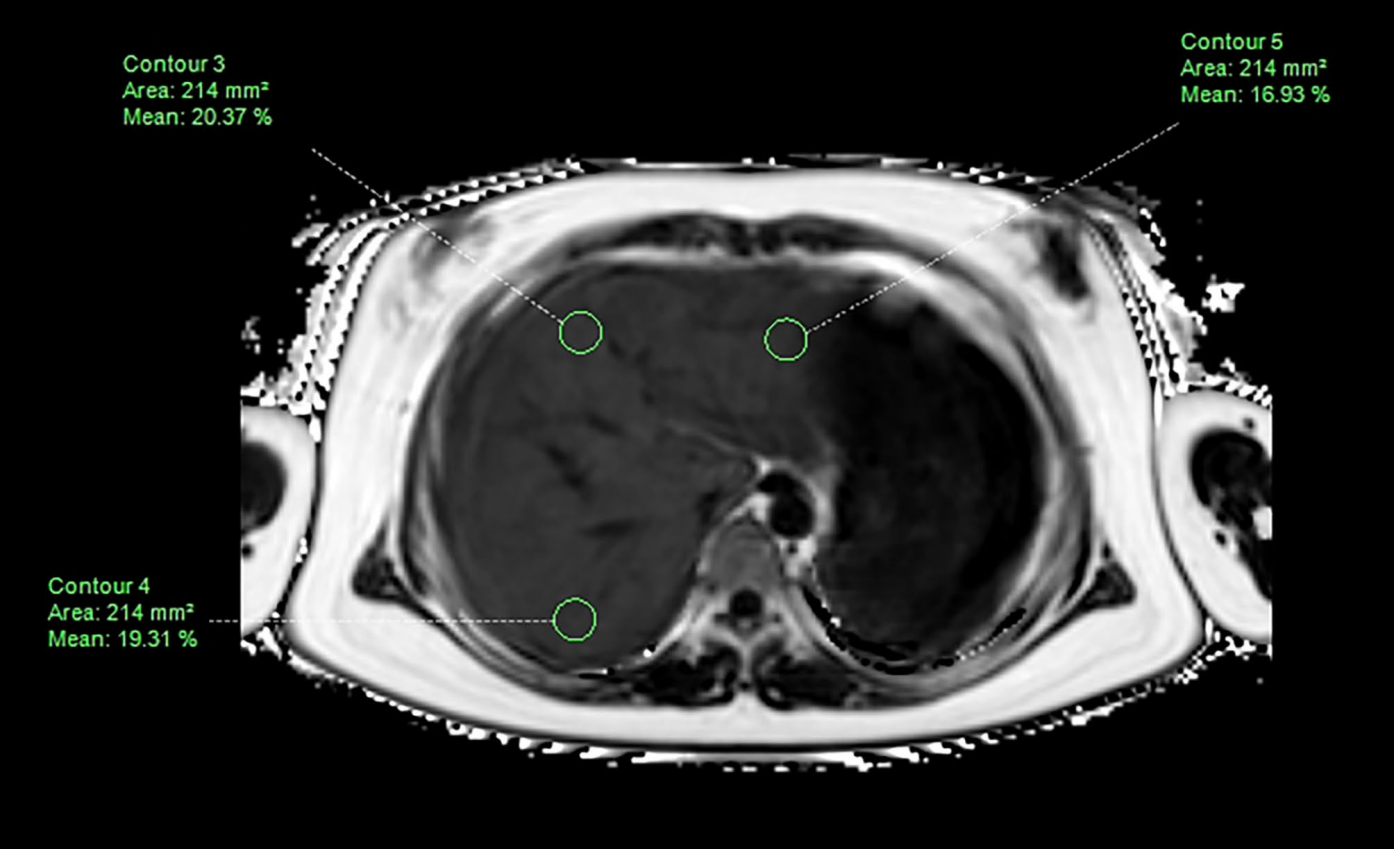
Liver fat content measurement with chemical shift-encoded magnetic resonance imaging

CSE-MRI datasets were processed using ISP version 7 software (Philips Healthcare, Best, the Netherlands) to generate confounder-corrected PDFF maps for PDFF measurements. Regions of interest (ROIs) were manually drawn on the axial image where the right branch of the portal vein enters the liver. The area of ROI was 214 ± 10 mm^2^. Three ROIs were identified in the liver parenchyma of the left, right anterior, and right posterior lobes, avoiding major bile ducts and vasculature, intrahepatic calcification, artefacts caused by the ribs, and gas in the lung or gastrointestinal tract (Fig. 1). If the left lobe was not visible at this level, the axial image with the largest transverse area of the left lobe was selected for measurement. The liver PDFF was calculated as the mean value of the three ROIs.

### Statistical analysis

Continuous variables were tested for normal distribution using the Shapiro–Wilk test and were represented as the mean ± standard deviation (SD) or median (interquartile range, IQR) according to the results. Categorical variables were expressed as numbers and percentages (n/%). The correlation between liver PDFF and anthropometric, biochemical, and sex hormonal measurements was examined using the Pearson’s correlation or Spearman’s correlation, as appropriate.

According to the current recommendations, patients with PCOS were categorised into the non-NAFLD group (PDFF ≤ 5.1%) and the NAFLD group (PDFF > 5.1%) based on PDFF values [14]. The independent-samples t-test or Mann–Whitney U test was used for intergroup comparisons. Receiver operating characteristic (ROC) curves, area under the curve (AUC), sensitivity, specificity, positive predictive value (PPV), and negative predictive value (NPV) were used to determine the cut-off values for diagnosing NAFLD and to evaluate diagnostic accuracy. Anthropometric, biochemical, and sex hormonal measurements were converted into categorical variables using their cut-off values. Univariate and multivariate logistic regression analyses were performed to evaluate the effects of anthropometric, biochemical, and sex hormonal measurements (used as independent variables) on NAFLD (used as the dependent variable) in overweight and obese patients with PCOS. The probability of NAFLD in overweight and obese women with PCOS was calculated according to the logistic formula: $\:{p}_{i}=\frac{1}{1+{e}^{-(\alpha\:+\beta\:{x}_{i})}}$. ROC curves were used to assess the accuracy of the combined indicators in screening NAFLD based on the true disease profile and disease probability calculated by the formula.

A two-tailed *p*-value of less than 0.05 was considered statistically significant. All statistical analyses were performed using the Statistical Program for Social Sciences (IBM Corp., Armonk, NY, US, Version 26.0).

## Results

### Relationships between liver PDFF and anthropometric, biochemistry, and sex hormonal measurements

A total of 87 overweight or obese patients with PCOS were recruited with a mean age of 29.4 ± 3.7 years (IQR, 27–31 years; range, 19–41 years), and the liver PDFF was 7.69% (3.93%, 14.80%). The anthropometric, biochemical, and sex hormonal measurements of all patients and their correlations with liver PDFF are summarised in Table 1. Liver PDFF was positively correlated with height, weight, BMI, AC, triglyceride, glucose, insulin, and FT and inversely correlated with HDL-C and SHBG.

**Table 1:** Correlation between liver PDFF and characteristics in overweight and obese PCOS patients

Variables	Statistical Value	Correlation coefficient^*^	*P*-value
Liver PDFF (%)	7.69 (3.93, 14.80)		
**General characteristics**			
Age (years)	29.4 ± 3.7	0.128	0.237
Height (m)	1.62 ± 0.05	-0.250	**0.020**
Weight (kg)	75.0 (70.0, 80.0)	0.320	**0.003**
BMI (kg/m^2^)	28.3 (26.2, 30.9)	0.456	< **0.001**
AC (cm)	91.0 (87.0, 98.0)	0.387	< **0.001**
**Biochemistry measurements**			
Triglyceride (mmol/L)	1.72 (1.27, 2.49)	0.413	< **0.001**
TC (mmol/L)	4.82 (4.20, 5.66)	0.189	0.080
HDL-C (mmol/L)	1.09 ± 0.21	-0.255	**0.017**
LDL-C (mmol/L)	3.08 ± 0.80	0.200	0.064
Glucose (mmol/L)	5.07 (4.81, 5.44)	0.416	< **0.001**
Insulin (pmol/L)	89.59 (64.23, 149.43)	0.445	< **0.001**
**Sex hormones**			
FSH (IU/L)	5.98 ± 1.76	-0.071	0.514
LH (IU/L)	7.71 (4.95, 11.99)	0.077	0.478
LH/FSH	1.28 (0.98, 2.05)	0.082	0.448
Estradiol (pg/mL)	49.91 (42.81, 61.30)	-0.012	0.912
Progesterone (ng/mL)	0.49 (0.27, 0.75)	-0.137	0.205
TT (pg/mL)	439.31 (314.40, 604.54)	0.050	0.645
FT (pg/mL)	9.02 ± 4.87	0.263	**0.014**
Prolactin (ng/mL)	10.44 (8.13, 13.35)	-0.082	0.451
SHBG (nmol/L)	26.6 (16.8, 45.7)	-0.464	< **0.001**

Data in normal distribution: mean ± SD; non-normal distribution: median (P25, P75)

^*^Spearman’s correlation was used

Bold font indicates statistical significance (*p* < 0.05).

AC, abdominal circumference; BMI, body mass index; FSH, follicle-stimulating hormone; FT, free testosterone; HDL-C, high-density lipoprotein cholesterol; LDL-C, low-density lipoprotein cholesterol; LH, luteinising hormone; PCOS, polycystic ovary syndrome; PDFF, proton density fat fraction; SD, standard deviation; SHBG, sex hormone-binding globulin; TC, total cholesterol; TT, total testosterone

### Comparisons of characteristics between overweight and obese PCOS patients with and without NAFLD

Overall, 28 patients (32.2%) had normal liver PDFF, whereas 59 patients (67.8%) had NAFLD. The liver PDFF in non-NAFLD and NAFLD groups was 3.24% ± 0.93% and 11.38% (7.63%, 17.97%), respectively. Weight, BMI, and AC were higher in patients with NAFLD than in those with normal liver PDFF. All biochemical measurements were statistically different between the groups, with higher triglycerides, TC, LDL-C, glucose, and insulin levels and lower HDL-C levels observed in the NAFLD group than in the non-NAFLD group. Among the numerous sex hormones, only FT and SHBG showed statistically significant differences between the groups; patients with NAFLD had higher FT and lower SHBG levels. Details are presented in Table 2.

**Table 2:** Comparison of characteristics between overweight and obese PCOS patients with and without NAFLD

Variables	Non-NAFLD (PDFF ≤ 5.1%, *n* = 28)		NAFLD (PDFF > 5.1%, *n* = 59)	*P*-value
Liver PDFF (%)	3.24 ± 0.93		11.38 (7.63, 17.97)	< **0.001**^**#**^
**General characteristics**				
Age (years)	28.6 ± 3.6		29.8 ± 3.8	0.173^*^
Height (m)	1.63 ± 0.04		1.62 ± 0.05	0.291^*^
Weight (kg)	71.6 ± 5.8		76.0 (71.8, 82.5)	**0.001** ^**#**^
BMI (kg/m^2^)	26.4 (25.4, 28.6)		29.4 (27.0, 31.6)	< **0.001**^**#**^
AC (cm)	88.8 ± 5.7		92.0 (88.8, 101.5)	**0.001** ^**#**^
**Biochemistry measurements**				
Triglyceride (mmol/L)	1.31 (0.95, 1.71)		1.87 (1.44, 2.69)	< **0.001**^#^
TC (mmol/L)	4.50 (4.02, 5.18)		5.18 ± 0.95	**0.011** ^#^
HDL-C (mmol/L)	1.16 ± 0.24		1.06 ± 0.19	**0.044** ^*^
LDL-C (mmol/L)	2.67 (2.39, 3.06)		3.22 ± 0.79	**0.012** ^#^
Glucose (mmol/l)	4.87 (4.51, 5.24)		5.16 (4.90, 5.48)	**0.006** ^#^
Insulin (pmol/L)	68.35 (49.97, 88.54)		114.46 (75.35, 163.90)	< **0.001**^#^
**Sex hormones**				
FSH (IU/L)	5.79 ± 2.17		6.07 ± 1.54	0.547^*^
LH (IU/L)	7.26 (3.54, 11.78)		7.80 (5.96, 12.90)	0.227^#^
LH/FSH	1.21 (0.88, 1.98)		1.44 (1.00, 2.12)	0.484^#^
Estradiol (pg/mL)	52.38 (32.80, 67.12)		48.79 (43.21, 58.71)	0.964^#^
Progesterone (ng/mL)	0.49 (0.33, 0.85)		0.49 (0.22, 0.74)	0.476^#^
TT (pg/mL)	353.06 (320.25, 582.95)		471.40 (311.10, 632.05)	0.493^#^
FT (pg/mL)	7.02 ± 4.63		9.97 ± 4.74	**0.008** ^*^
Prolactin (ng/mL)	10.53 (8.20, 13.22)		10.10 (7.78, 13.35)	0.630^#^
SHBG (nmol/L)	40.3 (26.8, 101.7)		23.6 (15.3, 31.6)	< **0.001**^#^

Data in normal distribution: mean ± SD; non - normal distribution: median (P25, P75)

^*^Independent-samples T-test was used; ^**#**^ Mann–Whitney U-test was used

Bold font indicates statistical significance (*p* < 0.05)

AC, abdominal circumference; BMI, body mass index; FSH, follicle-stimulating hormone; FT, free testosterone; HDL-C, high-density lipoprotein cholesterol; LDL-C, low-density lipoprotein cholesterol; LH, luteinising hormone; NAFLD, non-alcoholic fatty liver disease; PCOS, polycystic ovary syndrome; PDFF, proton density fat fraction; SD, standard deviation; SHBG, sex hormone-binding globulin; TC, total cholesterol; TT, total testosterone

### Diagnostic accuracy of anthropometric, biochemical, and sex hormonal measurements for NAFLD in overweight and obese patients with PCOS

The diagnostic accuracy of anthropometric, biochemical, and sex hormonal measurements for NAFLD in patients with PCOS is shown in Table 3. BMI, AC, triglyceride, insulin, and SHBG levels had a high AUC value (> 0.700). TC, LDL-C, glucose, and FT levels had lower AUC values (0.600–0.700). Insulin showed the highest specificity and PPV, whereas glucose showed the highest sensitivity and NPV.

**Table 3:** Diagnostic accuracy of indicators for NAFLD in overweight and obese PCOS patients

Variables	AUC (95% CI)	*P*-value	Cut-off value	Sensitivity	Specificity	PPV	NPV
BMI (kg/m^2^)	0.761 (0.658–0.864)	< **0.001**	26.8	79.7% (47/59)	64.3% (18/28)	82.5% (47/57)	60.0% (18/30)
AC (cm)	0.721 (0.609–0.833)	**0.001**	88.3	76.3% (45/59)	57.1% (16/28)	78.9% (45/57)	53.3% (16/30)
Triglyceride (mmol/L)	0.738 (0.620–0.856)	< **0.001**	1.57	71.2% (42/59)	75.0% (21/28)	85.7% (42/49)	55.3% (21/38)
TC (mmol/L)	0.670 (0.546–0.793)	**0.011**	4.67	69.5% (41/59)	64.3% (18/28)	80.4% (41/51)	50.0% (18/36)
HDL-C (mmol/L)	0.613 (0.481–0.744)	0.091	-				
LDL-C (mmol/L)	0.667 (0.549–0.784)	**0.012**	3.31	47.5% (28/59)	89.3% (25/28)	90.3% (28/31)	44.6% (25/56)
Glucose (mmol/l)	0.683 (0.554–0.812)	**0.006**	4.83	84.7% (50/59)	50.0% (14/28)	78.1% (50/64)	60.9% (14/23)
Insulin (pmol/L)	0.748 (0.643–0.853)	< **0.001**	111.35	52.5% (31/59)	92.9% (26/28)	93.9% (31/33)	48.1% (26/54)
FT (pg/mL)	0.674 (0.550–0.798)	**0.009**	7.60	66.1% (39/59)	71.4% (20/28)	83.0% (39/47)	50.0% (20/40)
SHBG (nmol/L)	0.737 (0.621–0.853)	< **0.001**	25.0	61.0% (36/59)	82.1% (23/28)	87.8% (36/41)	50.0% (23/46)

Bold font indicates statistical significance (*p* < 0.05)

AC, abdominal circumference; AUC, area under the curve; BMI, body mass index; CI, confidence interval; FT, free testosterone; HDL-C, high-density lipoprotein cholesterol; LDL-C, low-density lipoprotein cholesterol; NAFLD, non-alcoholic fatty liver disease; NPV, negative predictive value; PCOS, polycystic ovary syndrome; PPV, positive predictive value; SHBG, sex hormone-binding globulin; TC, total cholesterol

### Indicators for screening for NAFLD in overweight and obese patients with PCOS

The results of the logistic regression analysis are presented in Table 4. Univariate logistic regression analysis showed that BMI > 26.8 kg/m^2^, AC > 88.3 cm, triglyceride > 1.57 mmol/L, TC > 4.67 mmol/L, LDL-C > 3.31 mmol/L, glucose > 4.8 3 mmol/L, insulin > 111.35 pmol/L, FT > 7.6 pg/mL, and SHBG < 25 nmol/L were related to the occurrence of NAFLD in overweight and obese patients with PCOS. When all indicators were included in a multivariate logistic regression model, triglycerides, LDL-C, glucose, insulin, and SHBG contributed to the occurrence of NAFLD, and the Nagelkerke R^2^ of the final model was 0.572. However, BMI, AC, TC, and FT were excluded from the final model.

**Table 4:** Indicators for screening NAFLD in overweight and obese PCOS patients

	Univariate analysis			Multivariate analysis			
Variable	*β*	OR (95% CI)	*P-*value	*β*	OR (95% CI)	*P-*value	
BMI > 26.8 mmol/L	1.953	7.050 (2.594–19.158)	< **0.001**	—	—	—	Nagelkerke R^2^ 0.572
AC > 88.3 cm	1.455	4.286 (1.642–11.183)	**0.003**	—	—	—	Cox-snell R^2^ 0.409
Triglyceride > 1.57 mmol/L	2.003	7.412 (2.661–20.642)	< **0.001**	1.300	3.671 (1.010-13.348)	**0.048**	
TC > 4.67 mmol/L	1.411	4.100 (1.584–10.614)	**0.004**	—	—	—	
LDL-C > 3.31mmol/L	2.018	7.527 (2.047–27.674)	**0.002**	1.589	4.900 (1.009–23.797)	**0.049**	
Glucose > 4.83 mmol/L	1.715	5.556 (1.992–15.498)	**0.001**	1.314	3.720 (1.005–13.766)	**0.049**	
Insulin > 111.35 pmol/L	2.667	14.393 (3.128–66.223)	< **0.001**	2.054	7.799 (1.339–45.443)	**0.022**	
FT > 7.6 pg/mL	1.584	4.875 (1.827–13.005)	**0.002**	—	—	—	
SHBG < 25 nmol/L	1.974	7.200 (2.398–21.621)	< **0.001**	1.749	5.751 (1.433–23.077)	**0.014**	

Bold font indicates statistical significance (*p* < 0.05)

AC, abdominal circumference; BMI, body mass index; CI, confidence interval; FT, free testosterone; LDL-C, low-density lipoprotein cholesterol; NAFLD, non-alcoholic fatty liver disease; OR, odds ratio; PCOS, polycystic ovary syndrome; SHBG, sex hormone-binding globulin; TC, total cholesterol; *β*, regression coefficient

**Fig. 2:**
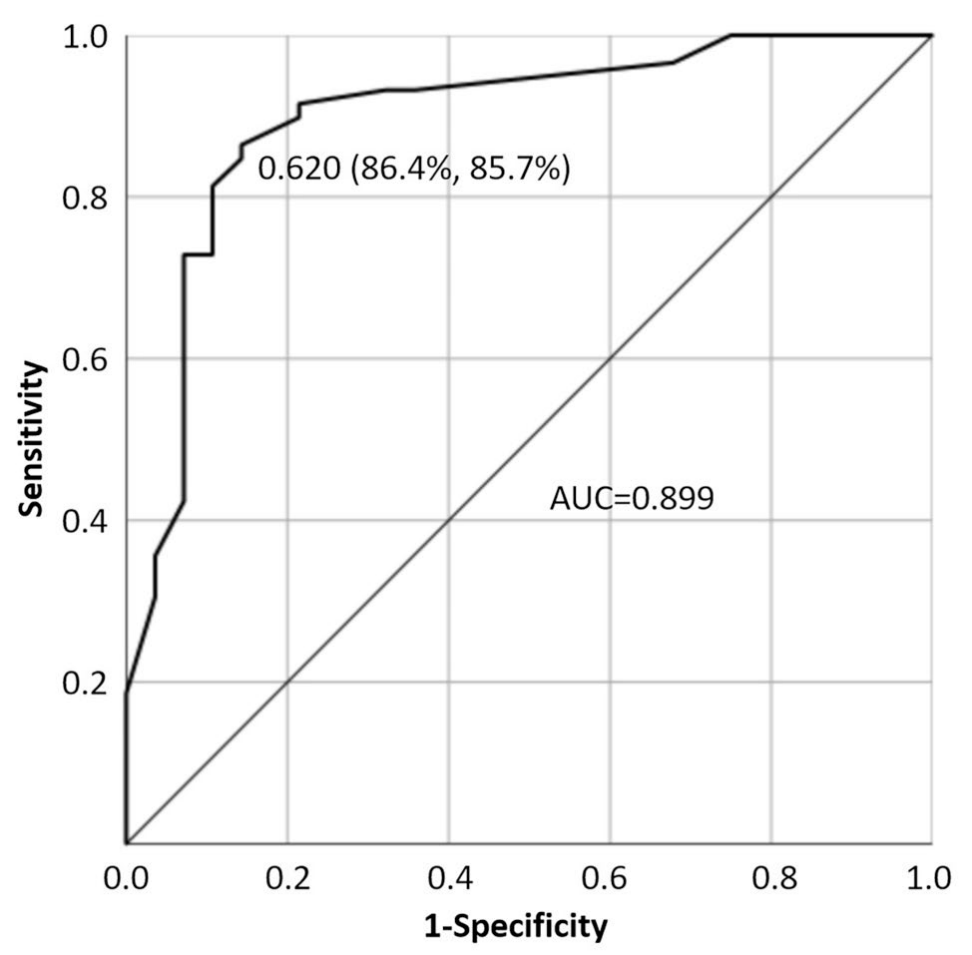
Receiver operating characteristic curve for non-alcoholic fatty liver disease screening according to the multi-indicator model. AUC, area under the curve

The probability equation of NAFLD in overweight and obese patients with PCOS was obtained as follows: $\:{p}_{i}=\frac{1}{1+{e}^{-(-2.258+1.300{{x}_{1}+1.589x}_{2}+1.314{x}_{3}+2.054{x}_{4}+1.749{x}_{5})}}$, where x_1_ is triglyceride (0,1), x_2_ is LDL-C (0,1), x_3_ is glucose (0,1), x_4_ is insulin (0,1), and x_5_ is SHBG (0,1). The ROC curve was established based on the true disease profile, and the disease probability was calculated using the formula (Fig. 2). The AUC was 0.899, with a 95% confidence interval (CI) of 0.823–0.974 (*P* < 0.001). The cut-off value of disease probability for screening NAFLD in overweight and obese patients with PCOS was 0.620, while the odds ratio (OR) was 38.250 with a 95% CI of 10.481–139.592 (*P* < 0.001). The sensitivity was 86.4% (51 of 59), specificity was 85.7% (24 of 28), PPV was 92.7% (51 of 55), and NPV was 75.0% (24 of 32).

## Discussion

The present study revealed the phenotype of NAFLD in overweight or obese young patients with PCOS and explored the anthropometric, biochemical, and sex hormonal measurements associated with the occurrence of NAFLD. The mean value of liver PDFF was 7.69% (3.93%, 14.80%) in overweight or obese PCOS patients in this study, with 67.8% of patients diagnosed with NAFLD. Both BMI and AC, representing overall and abdominal obesity, respectively, were significant risk factors for NAFLD, with thresholds of BMI > 26.8 kg/m² and AC > 88.3 cm. Metabolic risk factors associated with NAFLD included triglycerides > 1.57 mmol/L, TC > 4.67 mmol/L, LDL-C > 3.31 mmol/L, glucose > 4.83 mmol/L, and insulin > 111.35 pmol/L. Although many sex hormone abnormalities occur in PCOS, only FT (> 7.6 pg/mL) and SHBG (< 25 nmol/L) were associated with the occurrence of NAFLD. A multi-indicator model for NAFLD screening in patients with PCOS was established, which demonstrated high accuracy. This model included triglycerides, LDL-C, glucose, insulin and SHBG levels.

Despite numerous studies, the precise mechanism linking PCOS and NAFLD remains incompletely understood and is now generally believed to be related to hyperandrogenism, IR, obesity, and dyslipidemia [5, 17, 25]. These factors interact with each other, collectively affecting and being influenced by both PCOS and NAFLD, thus forming a vicious circle. Some researchers propose that NAFLD and PCOS are manifestations of metabolic syndrome in the liver and ovary, respectively [26].

IR is a fundamental characteristic of both PCOS and NAFLD and plays a pivotal role in their pathogenesis. IR, assessable through homeostatic model assessment for insulin resistance [27, 28], insulin sensitivity index [29], and quantitative insulin sensitivity check index [28], has been identified to be a risk factor for NAFLD in PCOS. Reduced insulin sensitivity diminishes the inhibitory effect of insulin on lipolysis in the peripheral adipose tissue, leading to a large influx of free fatty acids to and accumulating in the liver and subsequent hepatic accumulation [30]. Moreover, IR causes a compensatory increase in glucose and insulin levels, promoting de novo fat synthesis in the liver [31]. Additionally, IR is associated with liver damage and fibrosis in patients with PCOS [32, 33]. In the present study, patients with PCOS and NAFLD had higher glucose and insulin levels than did those without NAFLD. The overweight or obese patients with PCOS had a 5.6-fold and 14.4-fold higher risk of NAFLD when glucose > 4.83 mmol/L and insulin > 111.35 pmol/L, respectively.

Hyperandrogenism is not only the most probable aetiology of PCOS but also an independent risk factor for NAFLD in PCOS [34]. Patients with PCOS and hyperandrogenism have higher liver fat than those without hyperandrogenism and healthy controls [34]. Several animal model studies have explored the pathways through which hyperandrogenism affects the development and progression of NAFLD in PCOS, potentially through its impact on liver lipid metabolism, branch-chain amino acid metabolism, apoptosis, autophagy imbalance and inflammation [35–38]. In the present study, the occurrence of NAFLD in PCOS was associated with increased FT levels and decreased SHBG levels, which is consistent with previous findings [25, 27]. IR and hyperinsulinaemia in PCOS not only stimulate the ovaries to increase the production of androgens but also inhibit the synthesis of SHBG in the liver and increase free testosterone in the blood, leading to hyperandrogenaemia [15]. However, the liver PDFF showed no significant relationship with other sex hormones, including TT, FSH, and LH.

Overweight and obesity are prevalent among patients with PCOS and significantly contribute to the development of NAFLD, particularly abdominal obesity. Overweight and obese patients with PCOS are at a higher risk of developing NAFLD compared to non-obese patients with PCOS [25]. Although patients with PCOS and normal BMI were not included in our study, patients with NAFLD had higher BMI and AC than those without NAFLD. It has been reported that patients with PCOS and hyperandrogenaemia are more likely to develop NAFLD even in the absence of obesity; however, obesity exacerbates the prognosis [39]. The expansion of adipocyte volume, especially visceral adiposity, induces alterations in adipose tissue metabolism, disrupts the balance of proinflammatory cytokines and adipokines, promotes IR, and consequently, contributes to the development of NAFLD and hyperandrogenaemia [5, 40].

The lipid profile of PCOS is characterised by elevated triglyceride and LDL-C, along with decreased HDL-C [41, 42]. Dyslipidaemia has been linked to hyperandrogenaemia in PCOS, with the FAI positively correlating with TC, TG, and HOMA-IR and negatively correlating with HDL-C [43]. Hyperandrogenaemia has been hypothesised to directly affect hepatic LDL receptors, thereby making patients with PCOS more susceptible to dyslipidaemia and NAFLD [44]. Despite dyslipidaemia being prevalent in most patients with PCOS, our study found that patients with both PCOS and NAFLD exhibited more severe dyslipidaemia than those without NAFLD. Specifically, they had significantly higher levels of triglycerides, TC, and LDL-C levels and lower HDL-C levels. This finding aligns with the results of Vassilatou et al., who diagnosed hepatic steatosis in patients with PCOS using ultrasonography [25].

Given that hyperandrogenism, IR, obesity, and dyslipidaemia are associated with the development of NAFLD in patients with PCOS, various anthropometric, biochemical, and sex hormonal measurements have value in NAFLD screening. In the present study, the indicators with high diagnostic accuracy were BMI, insulin, triglycerides, SHBG, and AC, with the highest AUC of 0.761. However, none of the individual indicators achieved an AUC exceeding 0.8, indicating insufficient accuracy for single-indicator NAFLD screening. Consequently, we developed a multi-indicator model to screen for NAFLD screening in overweight or obese patients with PCOS using readily available anthropometric and laboratory parameters such as triglycerides, LDL-C, glucose, insulin, and SHBG levels. According to our findings, the probability of NAFLD occurrence in each patient with PCOS can be calculated using the multi-indicator model, and NAFLD will occur when the probability of incidence is greater than 0.620. This multi-indicator model demonstrated high diagnostic accuracy, with significantly higher AUC and OR compared to single indicators, along with high sensitivity, specificity, PPV, and NPV.

Ultrasound is a non-invasive imaging approach for diagnosing NAFLD. Although its diagnostic accuracy is affected by the operator’s technical proficiency and patient body type, and it lacks sensitivity to the early changes of NAFLD, its advantages of being easy to operate, cost-effective, and radiation-free have rendered it extensively utilized in the diagnosis of NAFLD [8, 45, 46]. However, according to the recommendations of the 2023 International Evidence-based Guideline for the Assessment and Management of Polycystic Ovary Syndrome [47], upper abdominal ultrasound is not a routine procedure for PCOS patients. Conversely, the anthropometric, biochemical, and sex hormonal parameters investigated in our study are essential indicators that should be routinely measured during the diagnosis and management of PCOS patients. The significance of our study on these indicators is not to completely replace the diagnostic value of ultrasound for NAFLD, but rather to use these routinely obtained indicators to screen for NAFLD in PCOS patients prior to upper abdominal ultrasound. This approach effectively reduces the unnecessary financial burden on patients and the time they spend waiting for examinations, thereby optimizing the clinical workflow and providing clinicians with valuable insights for early detection and timely intervention.

The cut-off values of most indicators in our study are stricter than the current normal reference standards. For example, while the current recommendation defines triglycerides greater than 1.70 mmol/L as abnormal, our study identified significance in diagnosing NAFLD with triglyceride levels greater than 1.57 mmol/L. Similar findings were observed in BMI (28 vs. 26.8 kg/m^2^), TC (5.18 vs. 4.67 mmol/L), LDL-C (3.34 vs. 3.31 mmol/L), glucose (6.10 vs. 4.83 mmol/L), insulin (174.00 vs. 111.35 pmol/L), and SHBG (34.3 vs. 25.0 nmol/L). This discrepancy may be attributed to the severe disease status of the overweight and obese patients with PCOS included in our study.

This study has a few limitations. First, being a cross-sectional study, it was unable to establish the dynamic relationship between PCOS, obesity, IR, hyperandrogenism, and NAFLD. Second, NAFLD encompasses a spectrum of liver pathologies, ranging from NAFL to NASH and cirrhosis, which can only be accurately distinguished by liver biopsy but not liver PDFF. Third, the multi-indicator model developed in this study requires further validation in a larger population to strengthen its generalizability. Nonetheless, this study revealed higher liver PDFF and a greater prevalence of NAFLD in overweight and obese patients with PCOS, which were associated with hyperandrogenism, obesity, and metabolic abnormalities. The proposed multi-indicator model offers a foundation for future research on the accurate screening of NAFLD using multiple parameters, not only in patients with PCOS but also in other populations.

## Conclusions

Overweight and obese patients with PCOS exhibit higher liver PDFF and a greater incidence of NAFLD. Among the anthropometric, biochemical, and sex hormonal measurements analysed, NAFLD occurrence in PCOS was associated with specific thresholds: BMI > 26.8 kg/m^2^, AC > 88.3 cm, triglycerides > 1.57 mmol/L, TC > 4.67 mmol/L, LDL-C > 3.31 mmol/L, glucose > 4.83 mmol/L, insulin > 111.35 pmol/L, FT > 7.6 pg/mL and SHBG < 25 nmol/L. A multi-indicator model, including triglyceride, LDL-C, glucose, insulin, and SHBG levels, has a high accuracy for screening NAFLD in overweight and obese patients with PCOS.

## Data Availability

The data supporting the findings of this study are available from the corresponding author upon reasonable request.

## Abbreviations

AC: Abdominal circumference
AUC: Area under the curve
BMI: Body mass index
CSE: Chemical shift-encoded
CI: Confidence interval
FAI: Free androgen index
FSH: Follicle-stimulating hormone
FT: Free testosterone
HDL-C: High-density lipoprotein cholesterol
IQR: Interquartile range
IR: Insulin resistance
LDL-C: Low-density lipoprotein cholesterol
LH: Luteinising hormone
MRI: Magnetic resonance imaging
NAFL: Non-alcoholic fatty liver
NAFLD: Non-alcoholic fatty liver disease
NASH: Non-alcoholic steatohepatitis
NPV: Negative predictive value
OR: Odds ratio
PCOS: Polycystic ovary syndrome
PDFF: Proton density fat fraction
PPV: Positive predictive value
ROC: Receiver operating characteristic
ROIs: Regions of interest
SD: Standard deviation
SHBG: Sex hormone-binding globulin
TC: Total cholesterol
TT: Total testosterone
